# Correlation between *Staphylococcus aureus* colonization and disease severity in atopic dermatitis: a systematic review and meta-analysis of randomized controlled trials

**DOI:** 10.3389/fimmu.2026.1820411

**Published:** 2026-05-07

**Authors:** Ruyi Jin, Mingyue Wang, Jincheng Ke, Xinghua Gao, Li Zhang

**Affiliations:** 1 Department of Dermatology, The First Hospital of China Medical University, Shenyang, China; 2 Key Laboratory of Immunodermatology, Ministry of Education, and National Health Commission; National Joint Engineering Research Center for Theranostics of Immunological Skin Diseases, Shenyang, China; 3 Department of Dermatology, The Second Affiliated Hospital of Xiamen Medical College, Xiamen, China

**Keywords:** atopic dermatitis, host–microbe interaction, meta-analysis, skin microbiome, *Staphylococcus aureus*

## Abstract

**Background:**

A positive association between *Staphylococcus aureus* (*S. aureus*) colonization and atopic dermatitis (AD) severity has been reported. However, the strength and consistency of this relationship remain unclear.

**Methods:**

We systematically searched PubMed, Embase, Cochrane Library, and Web of Science up to July 19, 2025. The reported correlation coefficients of studies have been extracted and converted into Fisher’s z-scores. The calculated values have been generated using a random-effects model in RevMan software and the final pooled result was converted to a correlation coefficient (*r*) with 95% confidence intervals. We tested the heterogeneity between the included studies using *I²*. We also provide sensitivity analyzes and subgroup.

**Results:**

Seven RCTs with 929 participants were included. The pooled analysis showed a moderate positive correlation between *S. aureus* colonization and AD severity (*r* = 0.42, 95%CI: 0.31–0.52). The observed heterogeneity was moderate but not significant (*I²* = 38%, *P* = 0.14). Sensitivity and subgroup analyzes supported the robustness of the main findings.

**Conclusions:**

*S. aureus* colonization is moderately associated with AD severity. However, it should not be interpreted as evidence of a direct causal relationship.

**Systematic Review Registration:**

https://www.crd.york.ac.uk/prospero/, identifier CRD420251104116.

## 1 Introduction

Atopic dermatitis (AD) is a chronic, recurrent inflammatory skin disease with intense pruritus (1). It affects up to 25% of children and 2-3% of adults (2). This disease causes serious deterioration in their quality of life. AD can be a result of various factors including genetic predisposition, immune system dysfunction, and environmental influences (3). There is also evidence on the role of skin microbiota which are not merely passive modifiers but can actively interact with the inflammatory process (4), potentially contributing to inflammatory processes through complex and context-dependent mechanisms, including microbial dysbiosis (5), modulation of innate and adaptive immunity (6), and bidirectional interactions whereby inflammation can in turn reshape microbial communities. For AD, *Staphylococcus aureus* (*S. aureus*) colonization is increased on lesional and non-lesional skin (4, 7). *S. aureus* can interfere with AD by cutaneous immune processes such as keratinocyte activation and type 2–skewed inflammation (8). The imperfect antimicrobial defense of AD also allows microbial overgrowth to provide an immunologic basis for studying microbe–severity relationships. Also, it is likely to increase the disease by producing virulence factors, breaking the skin barrier, and activating proinflammatory pathways (9).

Numerous studies have investigated the association between *S. aureus* colonization and AD severity. Some studies have reported moderate to strong correlations (10). Others report weak or no significant correlations between *S. aureus* and disease severity (11). Some analyzes focused on baseline conditions (12), and others evaluated changes occurring throughout therapeutic interventions (13). While conflicting findings have been reported, a more critical limitation lies in the lack of comparable quantitative evidence across studies. Many previous studies, particularly observational designs, are subject to substantial heterogeneity arising from differences in treatment exposure, disease stage, host-related characteristics, and microbiological assessment methods (14, 15). These variations not only contribute to inconsistent findings but also hinder the direct comparability of correlation estimates across studies.

As a result, it remains unclear to what extent *S. aureus* colonization is quantitatively associated with disease severity. Although heterogeneity cannot be entirely eliminated, randomized controlled trials (RCTs), with more structured study designs, predefined protocols, and standardized outcome assessments, may provide relatively more internally consistent and comparable data for evaluating this relationship (16).

To date, no meta-analysis has specifically focused on synthesizing correlation evidence derived from RCT settings. Therefore, we conducted a systematic review and meta-analysis based on RCTs. By pooling available evidence, this study aimed to clarify the association using evidence exclusively from RCTs.

## 2 Materials and methods

### 2.1 Search strategy

Following PRISMA criteria, this systematic review and meta-analysis was prospectively registered in PROSPERO (CRD420251104116). A comprehensive literature search was carried out in PubMed, Cochrane Library, Embase and Web of Science databases from inception to July 19, 2025. A search formula was set for each database using a combination of network words and keywords related to AD, *S. aureus*, relevance, severity, SCORing Atopic Dermatitis (SCORAD), Eczema Area and Severity Index (EASI), and randomized controlled trials. The full search strategies were provided in supplementary materials (**Supplementary file**). Further studies were located through manual screening of the reference lists of related papers. All citations and references were managed using EndNote software.

### 2.2 Selection of articles

#### 2.2.1 Eligibility criteria (based on PICOS)

The research question was defined according to the PICOS framework as follows:

Population(P): Patients with atopic dermatitis.
Exposure (E): *S. aureus* colonization (lesional skin).
Comparator (C): Not applicable.
Outcome (O): Disease severity scores (e.g., SCORAD, EASI) and their correlation with *S. aureus* colonization.
Study design (S): Randomized controlled trials (RCTs).

#### 2.2.2 Types of participants

Participants included individuals from RCTs involving AD patients, with or without healthy controls. Only the data of patients with AD were extracted, and the correlation between *S. aureus* colonization and disease severity was analyzed.

#### 2.2.3 Types of outcomes

Studies had to report a correlation coefficient (Pearson, Spearman, or repeated measures) between *S. aureus* colonization levels on lesional skin and severity scores such as SCORAD, EASI or oSCORAD.

### 2.3 Data extraction

Two researchers separately performed data extraction with standardized templates. The recorded data included study features (year of publication, author, country or region, and study design), participant details (sample size, age group, population type, and diagnostic criteria), and methodological features (sampling site, detection method, severity score, correlation type). For the results, we recorded correlation coefficients (*r* value), the corresponding *p* values, 95% confidence intervals, and whether the correlation is based on baseline or change-from-baseline values. Discrepancies between reviewers were primarily related to study characteristics, outcome data extraction, and classification of correlation types. These were resolved through discussion following predefined criteria, and, when necessary, adjudicated by a third reviewer. The level of agreement between reviewers was assessed using percentage agreement.

### 2.4 Quality assessment

The Cochrane Risk of Bias tool in RevMan(version5.4) was used to evaluate potential bias. Two authors conducted evaluations independently, and differences were resolved by consensus.

### 2.5 Data analysis

All reported correlation coefficients (*r*) were converted into Fisher’s Z values to stabilize variance. Given the limited number of included studies (n = 7) and the expected clinical and methodological heterogeneity across studies, a random-effects model was applied. The data were synthesized with a random-effects model (DerSimonian–Laird method) in Revman and converted to *r* with 95% confidence intervals. Analyses were conducted using RevMan version 5.4. Heterogeneity was assessed using Cochran’s Q test and the *I²* statistic. A *P*-value < 0.10 or *I²* > 50% (17) was considered indicative of moderate to substantial heterogeneity. Sensitivity was evaluated using leave-one-out analyzes.

Subgroup analyzes were conducted based on age groups, data types (baseline vs. change-from-baseline values), severity scoring methods and correlation methods. Given the limited number of included studies (n=7), subgroup analyzes were considered exploratory and should be interpreted with caution.

Because the number of included studies was limited (n = 7), which was lower than the commonly recommended threshold of 10, the Egger’s/Begg’s test analysis of publication bias was not performed (18).

## 3 Results

### 3.1 Literature search

**Figure 1:**
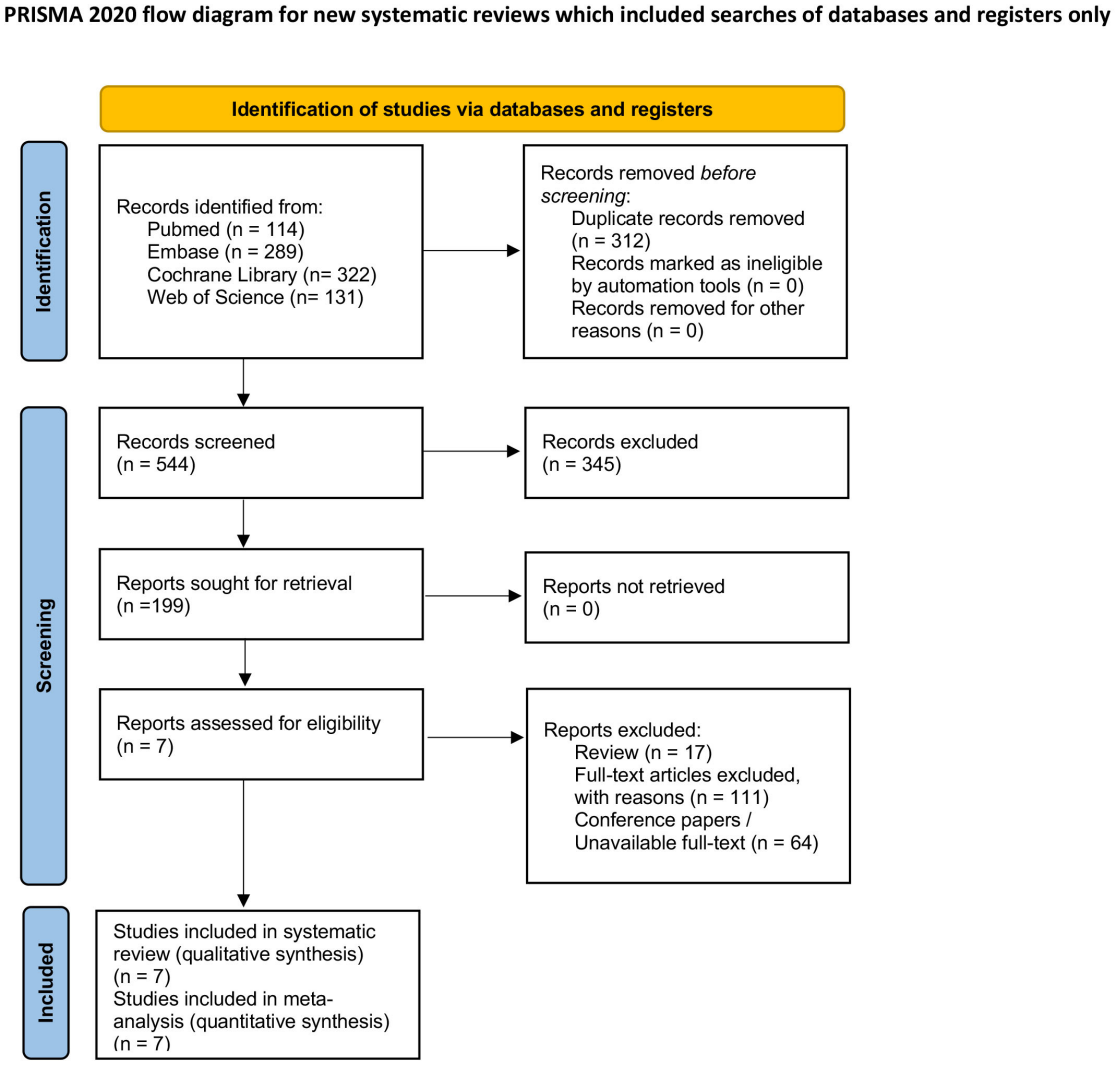
PRISMA flow diagram (38).

A total of 856 records were identified through searches of four electronic databases: PubMed, Cochrane Library, Embase and Web of Science (**Figure 1**). After removing 312 duplicates, 544 unique titles and abstracts were screened. Of these, 199 full-text articles were assessed for eligibility. 192 were excluded because they did not provide usable data (19), were protocols or early-phase trials, or lacked accessible outcomes. Ultimately, 7 RCTs met all inclusion criteria and were included in this meta-analysis. The study selection process is summarized in the PRISMA 2020 flow diagram.

### 3.2 Study characteristics

The seven trials were published between 2016 and 2023, enrolling a total of 929 participants (10, 12, 13, 20–23). Five studies included adults only (10, 12, 21–23), one focused on children (20), and one recruited both age groups (13). All studies used quantitative PCR (qPCR) to quantify *S. aureus* burden. Disease severity was assessed with EASI in four trials (10, 20, 21, 23), SCORAD in two (13, 22), and oSCORAD in one (12). Correlation analyzes were performed using Spearman’s rank correlation in most studies (10, 13, 20, 21, 23), with one study using Pearson’s correlation (12) and another using repeated-measures correlation (22). Key study details are summarized in **Table 1**.

**Table 1:** Characteristics of included studies .

No.	Author (year)	Country/region	Sample size	Population	Treatment	Severity scoring tool	Correlation type	Original r	Fisher’s z	SE	95% CI (z)	Data type
1	Smits et al. (2020)	Europe	7	Adults	Coal tar	EASI	Spearman	0.71	0.8872	0.5000	[-0.0928, 1.867]	Baseline
2	Gonzalez et al. (2016)	America	21	Children	TCS ± Bleach	EASI	Spearman	0.72	0.9076	0.2357	[0.4457, 1.370]	Baseline
3	Callewaert et al. (2020)	America、Canada	106	Adults	Dupilumab	EASI	Spearman	0.3792	0.3991	0.0985	[0.2060, 0.5922]	Baseline
4	Kolk et al. (2020)	Netherlands	36	Adults	Omiganan	oSCORAD	Pearson	0.409	0.4344	0.1741	[0.0932, 0.7756]	Baseline
5	Axt-Gadermann et al. (2021)	German	22	Children& Adults	Probiotic Bath	SCORAD	Spearman	0.62	0.7250	0.2294	[0.2754, 1.175]	Change-based
6	Simpson et al. (2023)	America	45	Adults	Dupilumab	SCORAD	Repeated Measures Correlation	0.39	0.4118	0.1543	[0.1094, 0.7142]	Repeated measures
7	Beck et al. (2023)	America、Europe、Japan	692	Adults	Tralokinumab	EASI	Spearman	0.3265	0.3390	0.0381	[0.2642, 0.4136]	Baseline

### 3.3 Methodological quality

**Figure 2:**
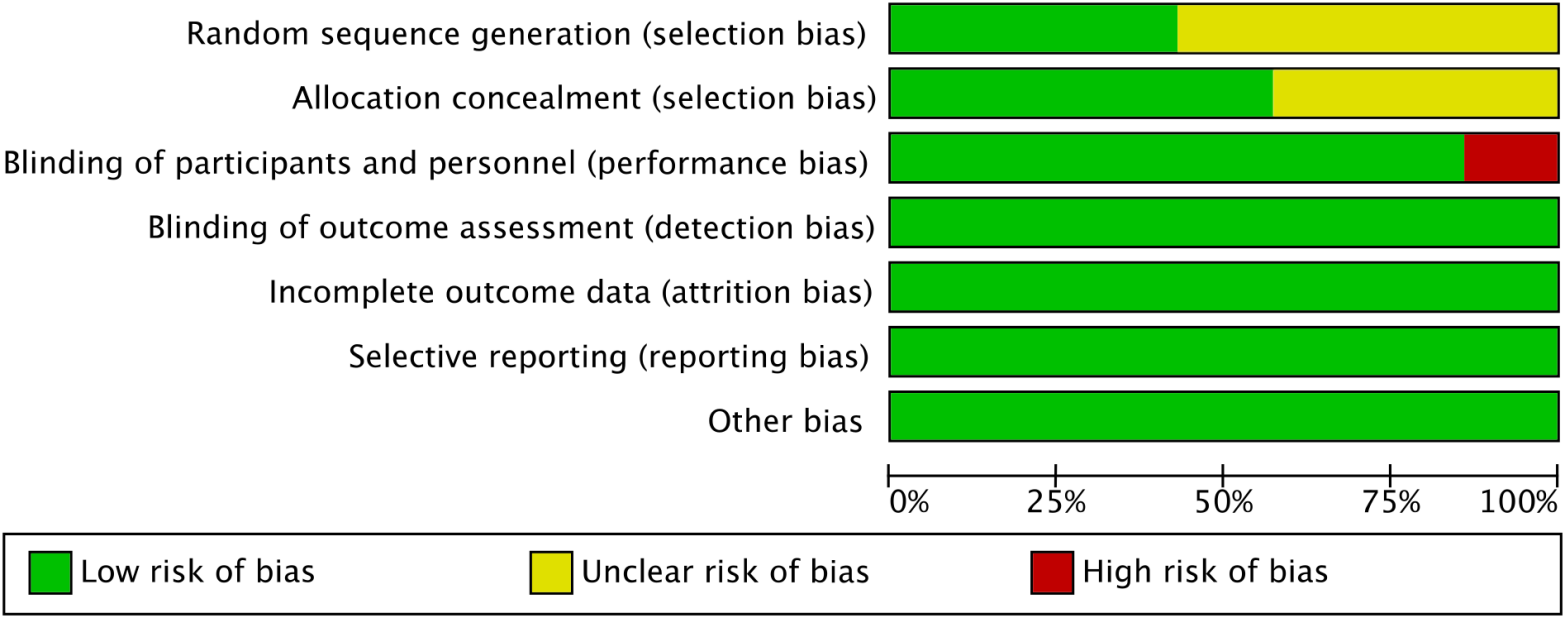
Risk of bias graph.

**Figure 3:**
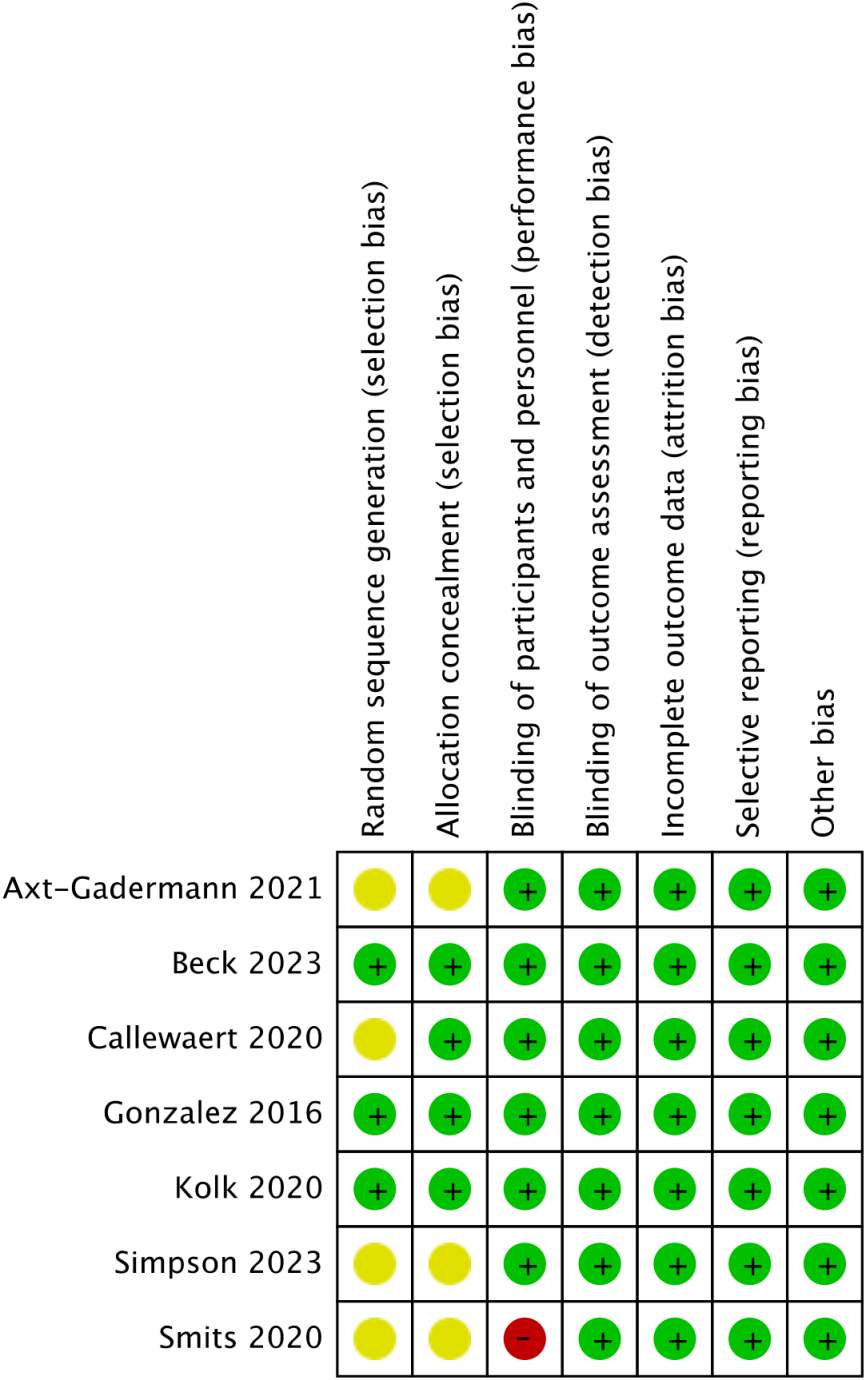
Risk of bias summary.

Using the Cochrane Risk of Bias tool(RoB 1), most domains were judged as low risk of bias, while a small proportion were rated as unclear or high risk, particularly in domains related to blinding (**Figures 2**, **3**). A formal GRADE assessment was not performed, as this meta-analysis focused on correlation coefficients rather than intervention effects, for which the applicability of the GRADE framework remains limited. These amendments have been updated in PROSPERO records. The method reported in this study is consistent with the final scheme.

The certainty of evidence was qualitatively assessed considering key domains including risk of bias, consistency of findings, and precision of estimates. Overall, the evidence was considered to be of moderate certainty, supported by consistent effect estimates across studies (*I²* = 38%) and relatively narrow confidence intervals, but limited by small sample size and potential residual confounding.

### 3.4 Main meta-analysis

**Figure 4:**
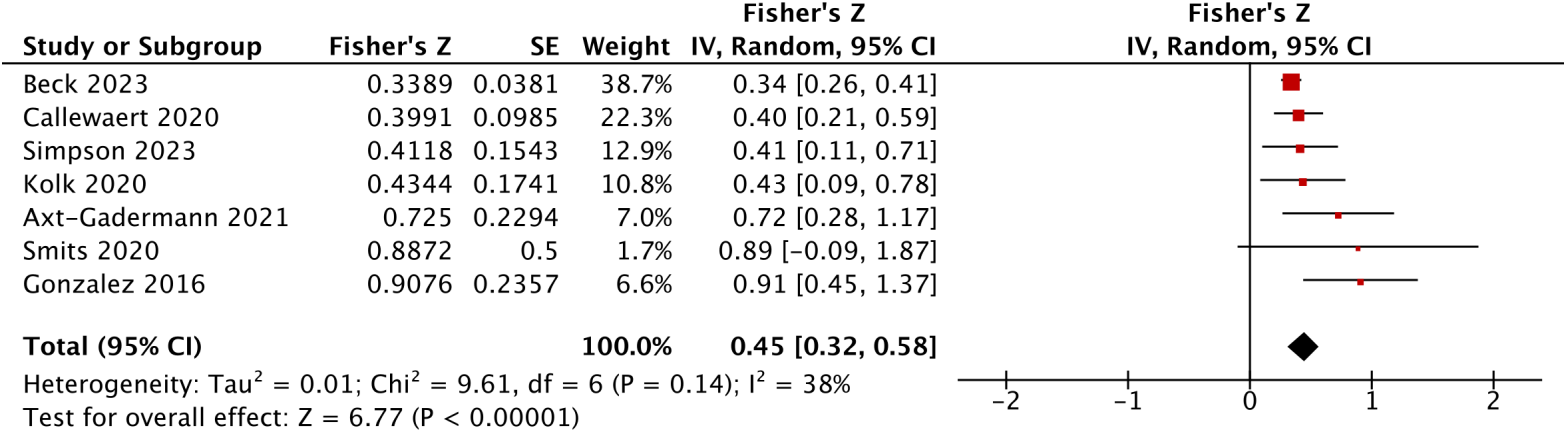
Overall pooled correlation between *S. aureus* colonization and AD severity across all included studies.

Considering the limited number of studies and potential clinical and methodological differences, a random effects model was applied. The result indicated moderate heterogeneity but no statistically significant heterogeneity based on Cochran’s Q test (*I²* = 38%, Cochran’s *Q* = 9.61, *P* = 0.14)(**Figure 4**). Although Cochran’s Q test was not statistically significant, this may be due to limited power given the small number of studies. The pooled analysis showed a moderate positive correlation between *S. aureus* burden and AD severity (Fisher’s *Z* = 0.45, 95%CI 0.32-0.58, *r* = 0.42, 95% CI 0.31–0.52).

### 3.5 Subgroup analyzes

Subgroup analyzes were performed to explore heterogeneity (**Table 2**).

**Table 2:** Summary of overall and subgroup effect sizes for the correlation between *Staphylococcus aureus* colonization and atopic dermatitis severity.

Category	Subgroup	k (studies)	Effect size (*r*)	95% CI	*I²* (%)	*P* (heterogeneity)
Overall	—	7	0.42	0.31–0.52	38	0.14
Age	Pediatric	1	0.72	0.42–0.88	—	—
	Adult	5	0.35	0.28–0.40	0%	0.76
	Pediatric and adults	1	0.62	0.27–0.82	—	—
Severity score	EASI	4	0.42	0.25–0.57	57%	0.07
	SCORAD	2	0.48	0.23–0.68	22%	0.26
	oSCORAD	1	0.41	0.09–0.65	—	—
Data type	Change-from-baseline (pooled)	2	0.48	0.23–0.68	22%	0.26
	Baseline cross-sectional (pooled)	5	0.41	0.27–0.52	44%	0.13
Correlation type	Spearman	5	0.46	0.29–0.60	58%	0.05
	Pearson	1	0.41	0.09–0.65	—	—
	Repeated-measures	1	0.39	0.11–0.61	—	—

r, correlation coefficient (back-transformed from Fisher’s Z); CI, confidence interval; k, number of studies; EASI, Eczema Area and Severity Index; SCORAD, Scoring Atopic Dermatitis; oSCORAD, objective SCORAD; *I²*, percentage of variability due to heterogeneity; “—” indicates not applicable.

#### 3.5.1 Age group

The pediatric study reported a strong correlation (*r* = 0.72, 95% CI 0.42–0.88). Five Adult-only studies showed a weaker pooled correlation (*r* = 0.35, 95% CI 0.28–0.40). The study including both pediatric and adult patients reported *r* = 0.62 (95% CI 0.27–0.82) (**Supplementary Figure 1**).

#### 3.5.2 Severity assessment method

Four studies using the EASI score showed *r* = 0.42 (95% CI 0.25–0.57) with moderate heterogeneity (*I²* = 57.0%). The two studies using SCORAD reported *r* = 0.48 (95% CI 0.23–0.68). The single oSCORAD study reported *r* = 0.41 (95% CI 0.09–0.65) (**Supplementary Figure 2**).

#### 3.5.3 Data type

Two studies analyzing change-from-baseline values showed *r* = 0.48 (95% CI 0.23–0.68). Five studies using baseline data showed *r* = 0.41 (95% CI 0.27–0.52) (**Supplementary Figure 3**).

#### 3.5.4 Analysis method

Five studies using Spearman’s rank correlation yielded *r* = 0.46 (95% CI 0.29–0.60). Pearson’s correlation, reported in a single study, had *r* = 0.41 (95% CI 0.09–0.65). Repeated-measures correlation, also from a single study, had *r* = 0.39 (95% CI 0.11–0.61) (**Supplementary Figure 4**).

As several subgroups were based on a single study, these estimates should be interpreted with caution and considered descriptive rather than inferential. No clear or consistent pattern of subgroup effects was observed; however, effect sizes in most subgroups generally fell within the moderate range of correlation. Furthermore, because most trials did not report treatment-specific correlations, it was not possible to distinguish treatment-related effects from baseline biological associations.

### 3.6 Sensitivity analysis

**Figure 5:**
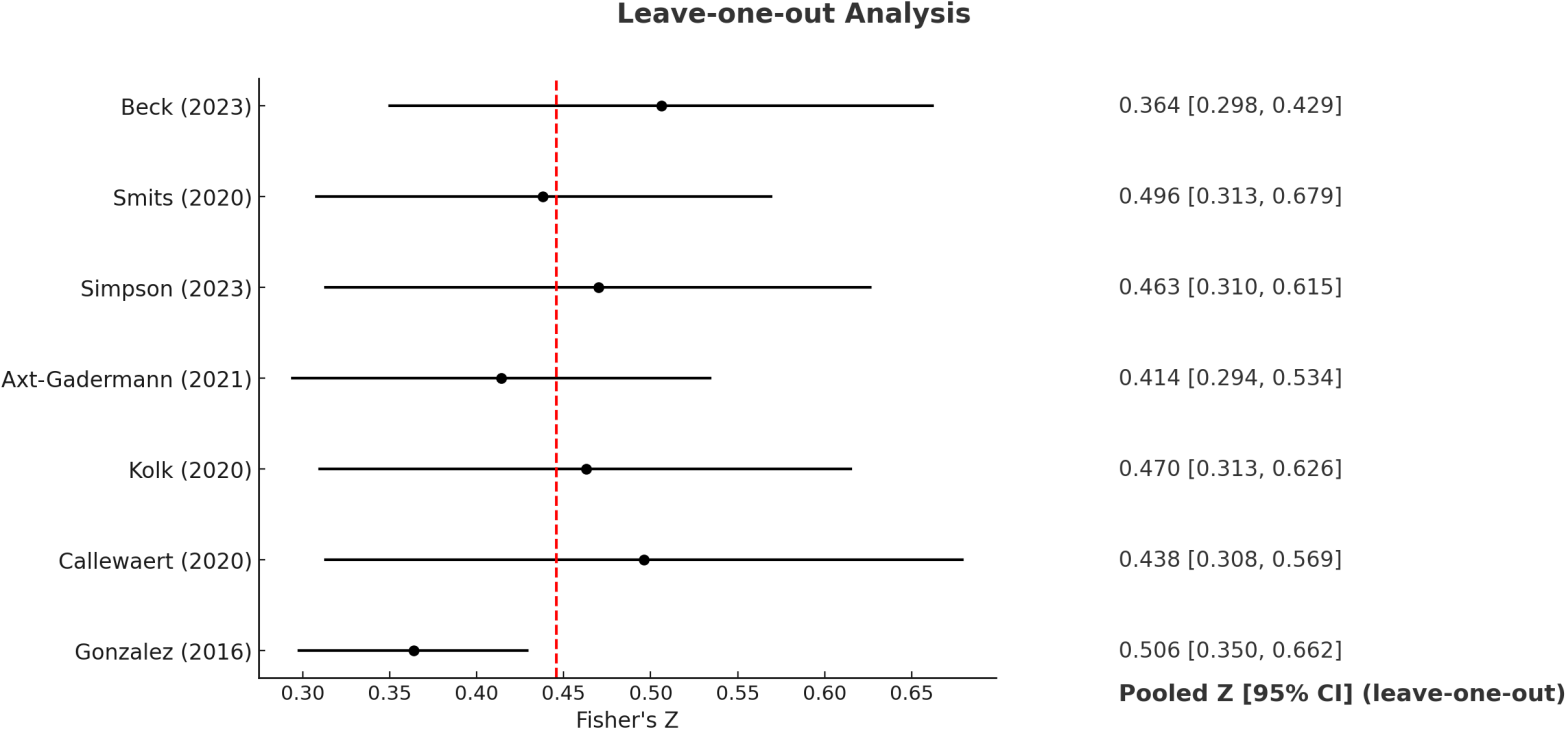
Leave-one-out analysis.

A leave-one-out sensitivity analysis was performed to assess the impact of individual studies on the pooled association. After sequentially excluding each study, the Fisher’s *Z* value ranged from 0.364 (95% CI 0.298–0.429) to 0.506(95% CI 0.350–0.662). Effect sizes remained within a similar range, indicating that no single study had a disproportionate influence on the overall results and supporting the robustness of the association between *S. aureus* colonization and AD severity (**Figure 5**).

## 4 Discussion

This meta-analysis of seven RCTs demonstrated a consistent positive association between *S. aureus* colonization in lesional skin and AD severity. The effect sizes remained broadly similar across scoring tools and analytic approaches, suggesting that the observed association is relatively robust and not driven by specific methodological choices.

From a clinical perspective, the pooled correlation (*r* = 0.42) indicates a moderate association, suggesting that *S. aureus* burden explains only part of the variability in disease severity. AD is a highly heterogeneous and multifactorial disease, with influenced by genetic susceptibility, skin barrier defects, immune dysregulation, environmental triggers, and the broader skin microbiome (24). Within this complex framework, *S. aureus* should be interpreted as a contributing factor rather than a primary driver. Its presence may reflect potential barrier damage or active inflammation, but it is not sufficient as an independent predictor of disease severity. This explanation emphasizes the importance of combining microbiological data with clinical and immunological parameters when evaluating disease status.

Mechanistically, experimental studies have shown that *S. aureus* can impair barrier function and amplify type 2 inflammation (25–27). In turn, IL-4/IL-13 signaling suppresses antimicrobial peptide production and weakens innate immune defenses, which is also a source of *S. aureus* persistence (8, 28, 29). This bidirectional interaction indicates that microbial colonization and immune dysfunction reinforce each other. However, as these mechanisms were not directly evaluated in the included trials, the observed associations should not be interpreted as evidence of causality.

Importantly, this non-causal interpretation can be further explained by structural confounding. Impaired skin barrier function can simultaneously facilitate greater *S. aureus* colonization and then lead to more severe disease manifestations (30), acting as a shared upstream factor. In this context, the observed correlation may not reflect a direct effect of bacterial load on disease activity, but rather the influence of this underlying factor on both variables.

Our findings are broadly consistent with previous observational studies and systematic reviews, which have also reported a positive association between *S. aureus* colonization and AD severity (31, 32). Similar associations have also been reported in more recent systematic reviews focusing on pediatric populations (32). However, many of these studies were based on cross-sectional or observational designs and may be more susceptible to confounding and bias. By restricting inclusion to RCTs, our analysis provides a more methodologically robust estimate of this association, although at the cost of a smaller number of included studies.

The stronger correlation observed in a single pediatric study may indicate age-related differences in host microbe interactions. Children typically have a more fragile skin barrier and a Th2-skewed immune profile (33, 34), which may increase susceptibility to microbial colonization and amplify its clinical impact. However, given the limited data, this observation should be interpreted with caution and warrants further investigation.

Several limitations should be considered. Firstly, only seven RCTs are included, which limits the statistical strength of our results. Secondly, the correlation coefficients come from published reports rather than individual participant data, precluding adjustment for potential confounders and limiting more detailed exploration of treatment-specific effects. Thirdly, all trials used qPCR to quantify *S. aureus*, which detects total bacterial DNA but cannot distinguish between viable and non-viable organisms (35). While this ensures methodological consistency across studies, it may lead to misestimation of the true burden of viable bacteria, thereby affecting the biological interpretation of the observed association (36).

From a clinical and research perspective, these findings suggest that S. aureus colonization should be interpreted primarily as a marker of disease activity or barrier dysfunction rather than a direct therapeutic target in the absence of infection, in line with current guidelines (37). Future research should prioritize longitudinal and interventional studies to clarify causal relationships, as well as broader investigations of the skin microbiome, including microbial diversity, interspecies interactions.

## 5 Conclusion

This systematic review and meta-analysis of seven RCTs showed a moderate positive correlation (*r* = 0.42, 95% CI 0.31–0.52) between *S. aureus* colonization on lesional skin and the severity of AD. This association should be interpreted as a marker of disease activity or barrier dysfunction, rather than evidence of direct causality. These findings highlight the importance of integrating microbiological, clinical, and immunological data when assessing disease status. Future research, especially longitudinal and interventional studies, is needed to clarify causal relationships and to further explore the broader role of the skin microbiome, including microbial diversity, interspecies interactions.

## Data Availability

The original contributions presented in the study are included in the article/**Supplementary Material**. Further inquiries can be directed to the corresponding author.
