# Supplementary material for: Correlation between Staphylococcus aureus colonization and disease severity in atopic dermatitis: a systematic review and meta-analysis of randomized controlled trials
Source: Front Immunol. 2026 May 7;17:1820411. doi: 10.3389/fimmu.2026.1820411 (PMC13189906; doi:10.3389/fimmu.2026.1820411)
Supplement: Supplementary file 1 [file DataSheet1.docx]

Supplementary Material

# Supplementary Data

**Search strategy**

1.PubMed

(("Dermatitis, Atopic"[Mesh] OR "Eczema"[Mesh] OR "atopic dermatitis"[tiab] OR "atopic eczema"[tiab] OR "AD"[tiab] OR "AE"[tiab])
AND("Staphylococcus aureus"[Mesh] OR "staphylococcus aureus"[tiab] OR "S. aureus"[tiab]
OR "staphylococcal"[tiab] OR "coagulase positive staphylococcus"[tiab]
OR "golden staph"[tiab] OR "SA"[tiab]
OR ("bacterial culture"[tiab] OR "qPCR"[tiab] OR "16S sequencing"[tiab]))
AND
("Treatment Outcome"[Mesh] OR "therapeutic"[tiab] OR "treatment effect"[tiab]
OR "clinical response"[tiab] OR "clinical improvement"[tiab]
OR "EASI"[tiab] OR "SCORAD"[tiab] OR "IGA"[tiab]
OR "severity score"[tiab] OR "severity index"[tiab]
OR "severity reduction"[tiab] OR "symptom improvement"[tiab])
AND
("Randomized Controlled Trial"[Publication Type] OR "randomized"[tiab] OR "randomised"[tiab]
OR "RCT"[tiab] OR "controlled trial"[tiab] OR "clinical trial"[tiab]
OR ("cohort study"[tiab] AND "prospective"[tiab])))

2.Cochrane Library

#1 MeSH descriptor: [Dermatitis, Atopic] explode all trees
#2 MeSH descriptor: [Eczema] explode all trees
#3 (atopic dermatitis or atopic eczema or AD or AE):ti,ab,kw
#4 #1 or #2 or #3
#5 MeSH descriptor: [Staphylococcus aureus] explode all trees
#6 (staphylococcus aureus or S. aureus or staphylococcal or coagulase positive staphylococcus):ti,ab,kw
#7 #5 or #6
#8 MeSH descriptor: [Treatment Outcome] explode all trees
#9 (therapeutic or clinical response or EASI or SCORAD or IGA):ti,ab,kw
#10 #8 or #9
#11 #4 and #7 and #10

3.Embase

#1 'atopic dermatitis'/exp OR 'eczema'/exp
#2 'atopic dermatitis':ab,ti OR 'atopic eczema':ab,ti OR 'AD':ab,ti OR 'AE':ab,ti
#3 #1 OR #2
#4 'staphylococcus aureus'/exp OR 'methicillin resistant staphylococcus aureus'/exp
#5 'staphylococcus aureus':ab,ti OR 's aureus':ab,ti OR 'staphylococcal':ab,ti OR 'coagulase positive staphylococcus':ab,ti
#6 #4 OR #5
#7 'treatment outcome'/exp OR 'therapeutics'/exp
#8 'therapeutic':ab,ti OR 'clinical response':ab,ti OR 'EASI':ab,ti OR 'SCORAD':ab,ti
#9 #7 OR #8
#10 'randomized controlled trial'/exp OR 'controlled clinical trial'/exp
#11 'randomized':ab,ti OR 'randomised':ab,ti OR 'RCT':ab,ti OR 'controlled trial':ab,ti
#12 #10 OR #11
#13 #3 AND #6 AND #9 AND #12

4.Web of Science

#1 TS=("atopic dermatitis" OR "atopic eczema")
#2 TS=("staphylococcus aureus" OR "S. aureus")
#3 TS=(treatment OR therapy OR therapeutic)
#4 #1 AND #2 AND #3
#5 DT=(Clinical Trial)
#6 #4 AND #5

# Supplementary Figures


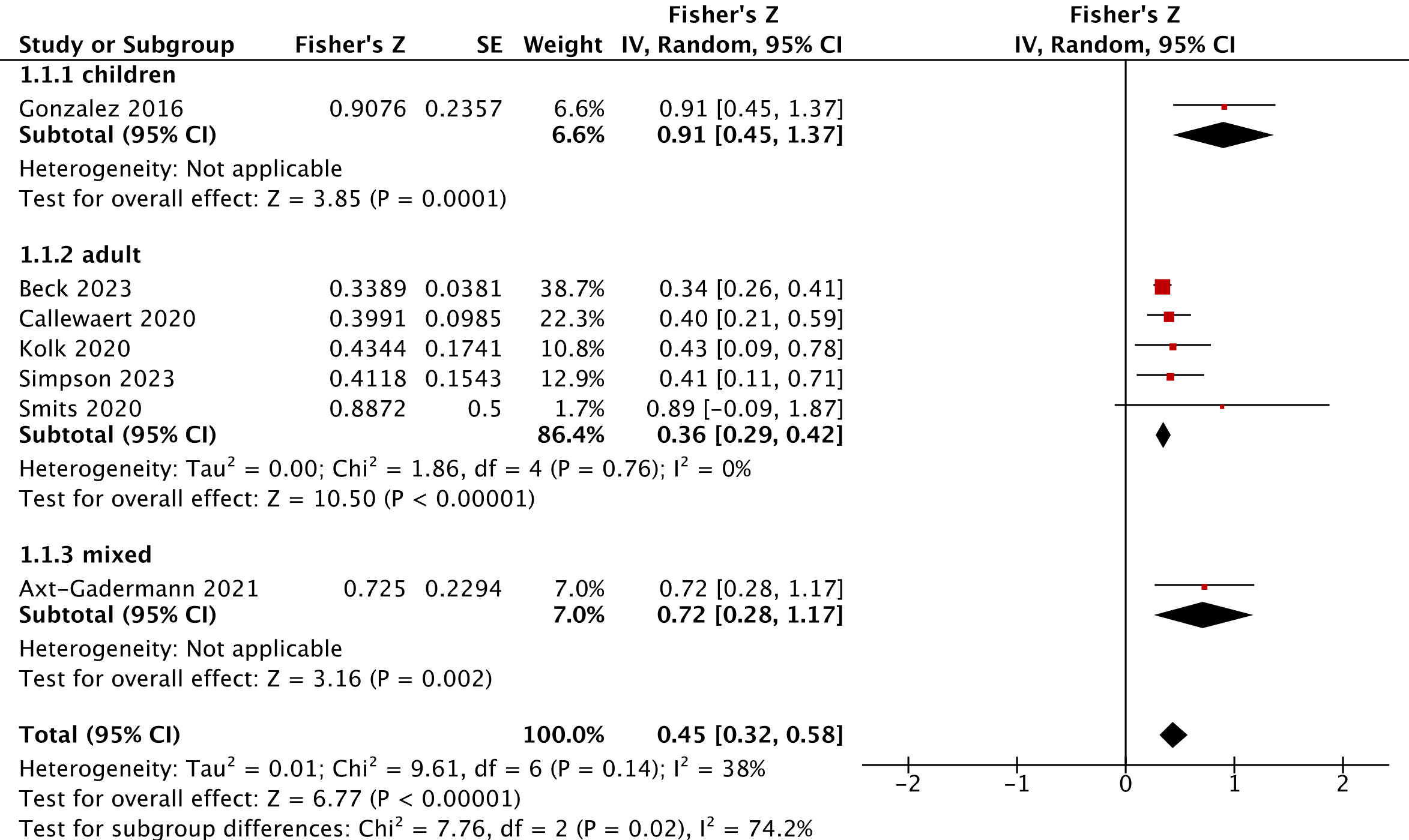


**Supplementary Figure 1.** Subgroup analysis by age.


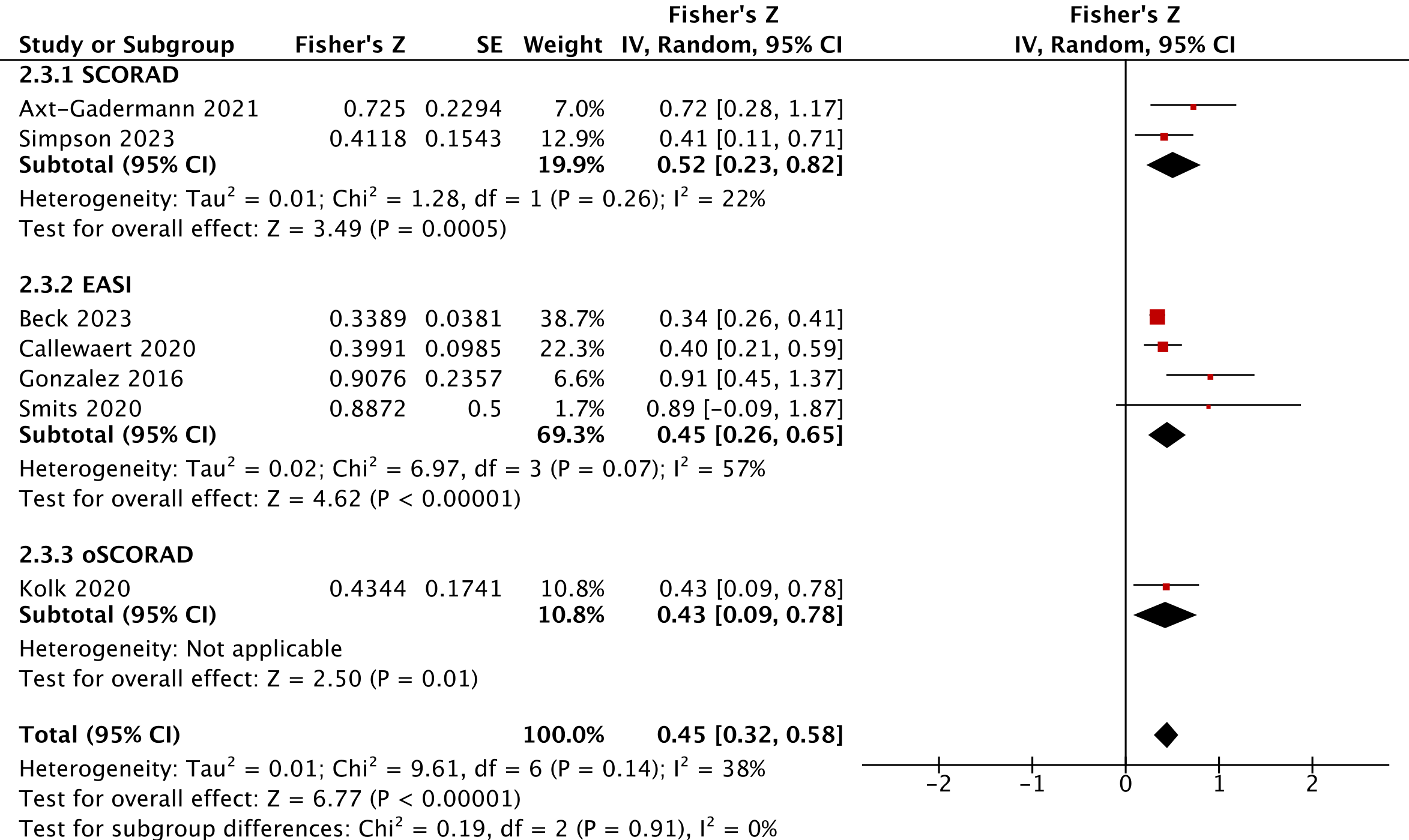


**Supplementary Figure 2.** Subgroup analysis by severity scoring method.


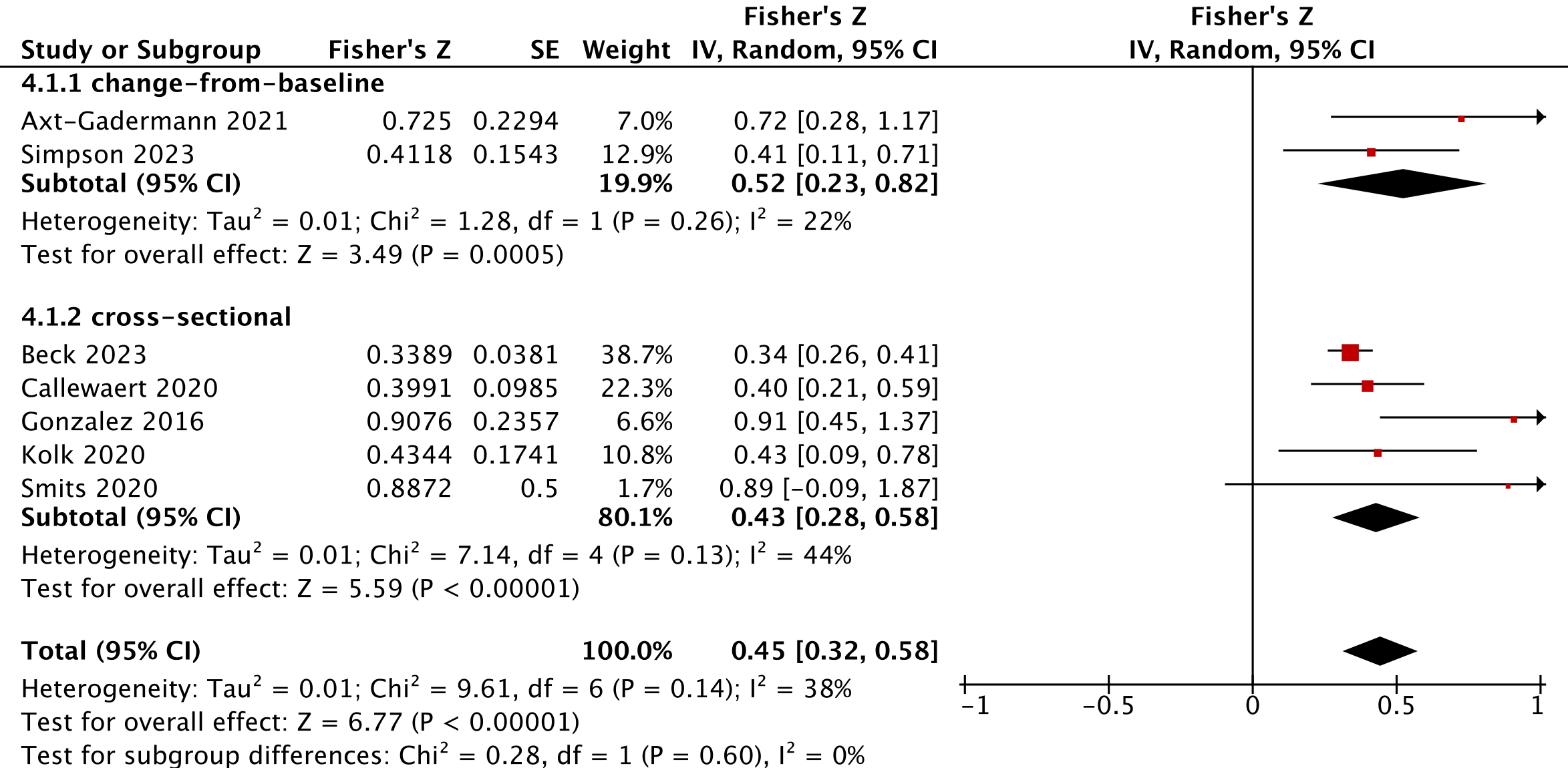


**Supplementary Figure 3.** Subgroup analysis by data type.


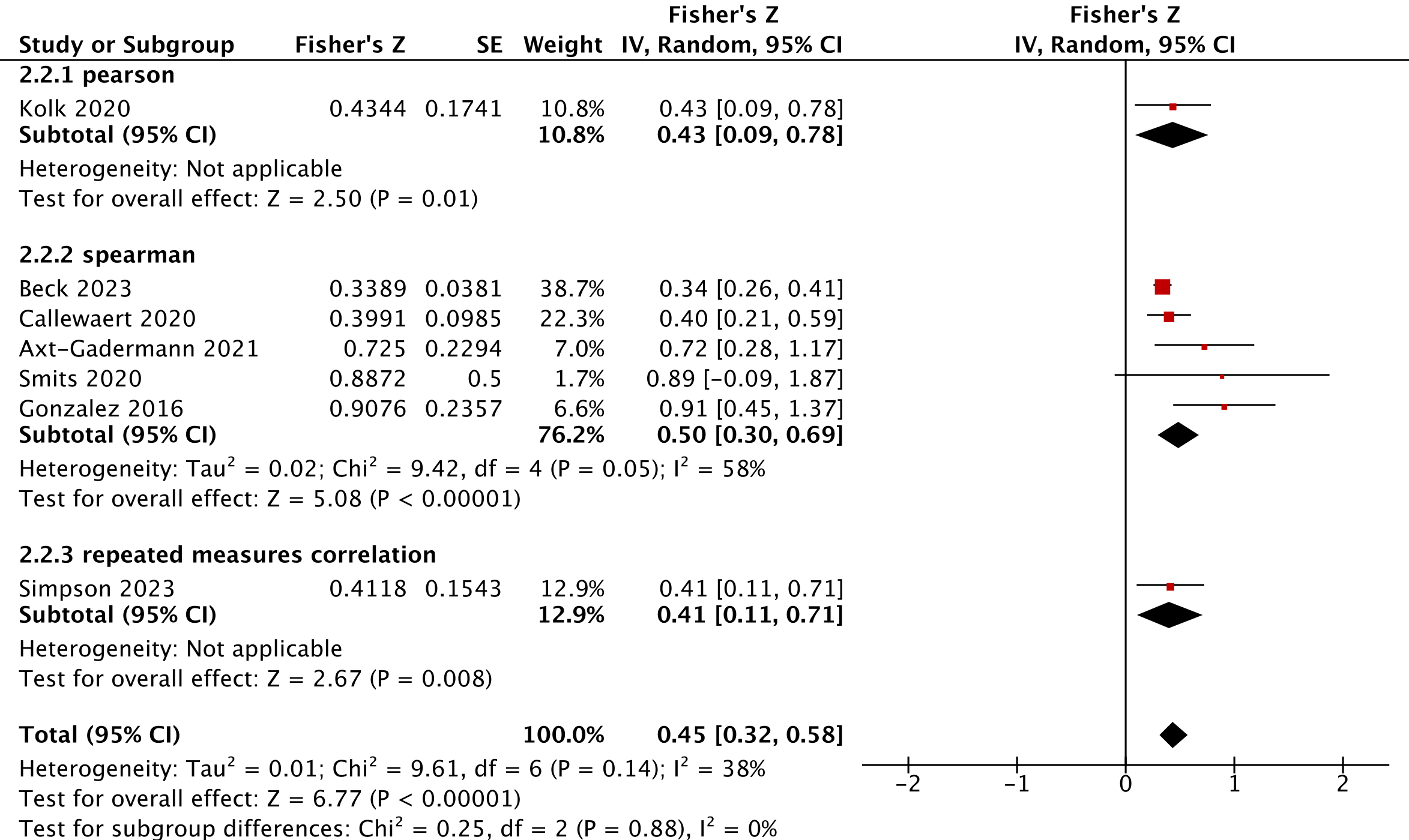


**Supplementary Figure 4.** Subgroup analysis by correlation method.
